# Giant Basal Cell Carcinoma of the Lateral Neck: A Case Study

**DOI:** 10.7759/cureus.44487

**Published:** 2023-08-31

**Authors:** Alec M Bryson, Nicole Dominiak, Patrick W Frank

**Affiliations:** 1 Medical Education, College of Medicine and Life Sciences, University of Toledo, Toledo, USA; 2 Pathology, College of Medicine and Life Sciences, University of Toledo, Toledo, USA

**Keywords:** peri-auricular invasion, giant basal cell carcinoma, cadaver case report, basal cell carcinoma histopathology, local invasion, head and neck neoplasms, basal cell carcinoma

## Abstract

A giant basal cell carcinoma (GBCC) is a rare variant of basal cell carcinoma (BCC) that is larger (>5 cm) and more aggressive. While BCC is usually surgically excised as a small, local tumor, cases of GBCC represent a considerable portion of BCC malignancies and mortality. The growth of GBCC is hypothesized to be multifactorial, and due to the successful treatment of BCC, available data is limited. We present a case of GBCC found during routine post-mortem dissection in a 92-year-old male cadaver. The neoplasm showed predilection to periauricular soft tissue invasion, despite demonstrating high-risk characteristics for metastasis. Microscopic analysis demonstrated an infiltrative growth pattern and neurotropism. Perineural spread could be observed on gross dissection, indicating a worse prognosis, but there was no evidence of lymphatic or hematogenous spread. This is most likely due to the stromal dependence of BCC. Local invasion of the primary tumor likely compromised head and neck function, but there was no secondary tumor evidence. There were no histopathological findings that indicate an aggressive growth or metastatic transformation of the tumor. Therefore, while a conclusion about duration cannot be made due to the anonymity of the cadaver, duration of growth likely was a significant factor in mortality.

## Introduction

Basal cell carcinoma (BCC) is the most common skin cancer, with a higher risk of development on sun-exposed areas such as the head and neck. BCC is a slow-growing (<1 mm per year), locally destructive tumor. BCC has a stromal dependence, which explains why metastasis is rare (<1%) [1-2]. Surgical excision is often curative. However, tumor diameter >5 cm is defined by the American Joint Committee on Cancer as a giant basal cell carcinoma (GBCC), a rare, aggressive variant (<0.5%) that accounts for a substantial portion of malignant and fatal BCC [3]. Compared to BCC, GBCC is more likely to metastasize and significantly damage local soft tissue, challenging clinical management. Growth of GBCC is hypothesized to be multifactorial, with neglect being the most commonly cited contributor [3-4]. Other factors may include recurrence of a previous tumor, immunosuppression, and genetic factors of both the tumor and/or patient. Because of the curative treatment of BCC, GBCC is underreported in the literature. There are multiple theories about whether or not GBCC has certain histopathological features predisposing it to faster growth and spread [3]. However, there is a lack of definitive conclusions on whether these theories hold any value. This case details a cadaver dissection of a GBCC with the goal of understanding the growth of the neoplasm and the role of the neoplasm in the patient’s death.

## Case presentation

This case originates from the dissection of a 92-year-old, white, non-smoking, male priest who donated his body to the University of Toledo College of Medicine and Life Sciences. The body was initially used for routine dissection of the body cavities and the extremities in the gross anatomy lab. Due to the anonymity of the body donation program, a full clinical history is not available. The cause of death was reported as a “malignant neoplasm of soft tissue of the (left) neck.” Secondary contributing causes include otorrhagia of the left ear and anemia. In the state of Ohio, cause of death is provided by the physician who oversaw the patient’s care for the illness or condition that resulted in death.

**Figure 1:**
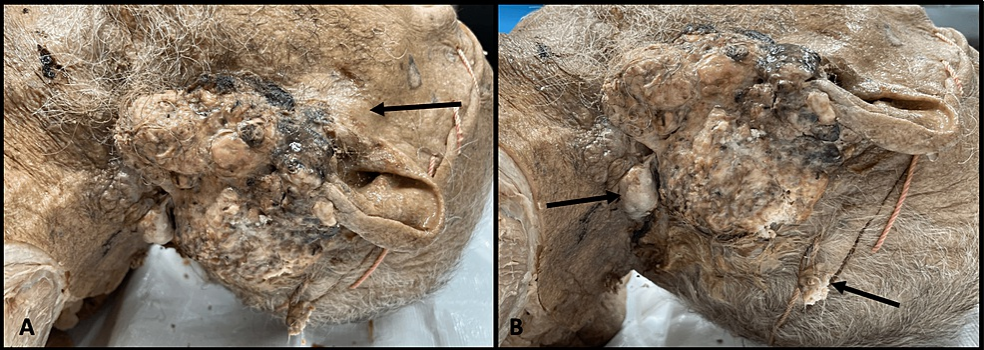
Gross appearance of the tumor (A) Appearance of the tumor before dissection showing invasion into the external ear and growth under the skin into the parotid gland (arrow). (B) View of the postauricular area with polyp-like growths (arrows). The tumor measured 10.8 cm in the cranial-caudal direction and 7.8 cm in the anterior-posterior direction.

The neoplasm was found on the left periauricular area, measuring 7.8 x 10.6 cm in diameter with growth invading the lobule of the ear and the parotid gland. The neoplasm had areas of dark brown and black discoloration and a firm, nodular texture with a few polyp-like growths posteriorly (Figure 1). Firmness was palpated deep to the skin and into the left parotid gland.

**Figure 2:**
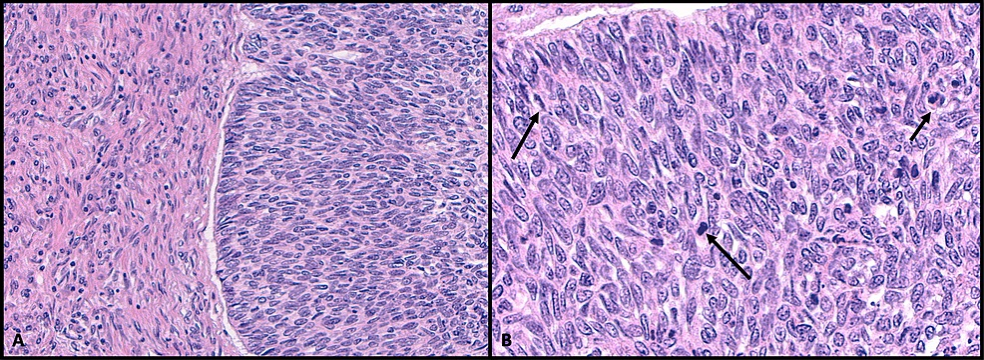
Histopathological pattern observed from isolated tumor specimen (A) Sample of the tumor showing peripheral palisading and tumor-stromal clefting, characteristic histologic features of basal cell carcinoma (20x). (B) High-power view (40x) of mitoses consistently seen in the nests and nodules of basal cell carcinoma (arrows).

A head and neck dissection was performed, and local tissue invasion was observed. Growth occurred through the sternocleidomastoid muscle (SCM), with a marble-sized hard growth deep to SCM and anteriorly attached to the posterior belly of the digastric muscle. The levator scapular, splenius capitus, trapezius, and scalene muscles were all intact. The tumor also invaded the left parotid gland, which was 2.3 x 1.8 cm, smaller in dimension than the right parotid gland. Multiple portions of the tumor were isolated, demonstrating a basaloid epithelial tumor with characteristic peripheral palisading and cleft formation between the tumor nests and stroma on microscopic analysis (Figure 2A).

The neoplasm was also observed growing around the external jugular vein, the retromandibular vein, the external carotid artery, and the posterior auricular artery. The vasculature appeared intact and not compromised on gross examination. Microscopic analysis of portions of the common and external carotid arteries, including the bifurcation, showed no evidence of infiltration, pre-mortem thrombus, or atherosclerosis.

**Figure 3:**
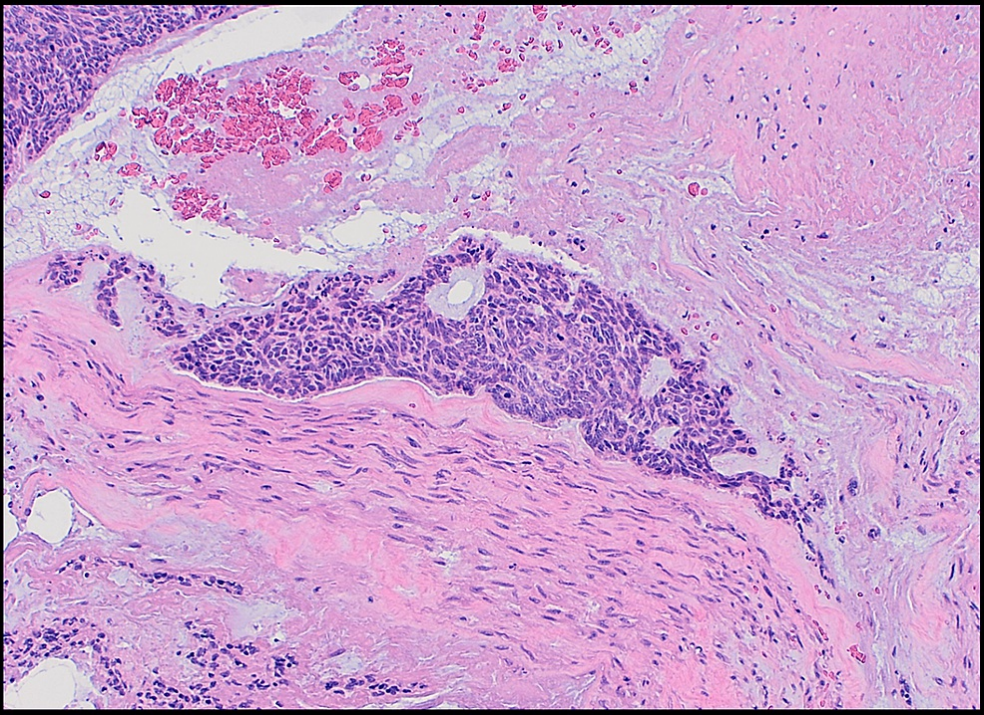
Example of neurotropism observed on microscopic analysis One of many foci of neurotropism within the carcinoma. There is a nest of basal cell carcinoma coursing alongside a nerve. This increases the probability of the presence of perineural invasion elsewhere in the tumor (10x).

The following nerves were identified coursing through the tumor: facial, accessory, auriculotemporal, lesser occipital, and greater auricular. The facial nerve was observed entering the tumor distally to the stylomastoid foramen and exiting as branches in the parotid gland. The tumor extended along the facial nerve toward the stylomastoid foramen. Perineural spread (PNS) and multiple areas of neurotropism were seen on histology (Figure 3), increasing the likelihood of perineural invasion (PNI).

**Figure 4:**
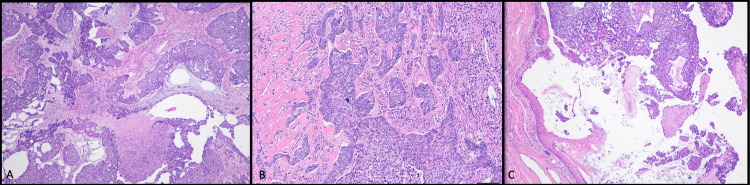
An infiltrative growth pattern observed in multiple sections of tumor specimen (A) Low power (2x) overview of basal cell carcinoma with nests of atypical basaloid cells coursing through stroma and an area of tumor necrosis. (B) Infiltrative (aggressive) pattern of growth present in the tumor (4x). (C) Low power view (2x) of the basal cell carcinoma involving the squamous-lined external auditory canal.

Dried blood was found in the external auditory canal (EAC) and on the tympanic membrane, and invasion of the EAC soft tissue was seen on histology (Figure 4C). There was no evidence of middle ear damage, tympanic membrane perforation, ossicle damage, or temporal bone invasion. The rest of the head and neck revealed no abnormalities, neoplasms, or signs of malignancy. The brain showed no signs of metastasis, ischemia, or gross hemorrhage. Regional and mediastinal lymph nodes were unremarkable. The thorax and abdomen were unremarkable.

## Discussion

While BCC is the most common skin cancer, metastasis of BCC carries a poor prognosis. Being a rare occurrence, many current hypotheses are limited by the lack of data in the current literature. Therefore, the growth rate of a GBCC has been hypothesized to be multifactorial. It is currently unclear if a certain histopathology subtype or other tumor characteristic contributes to an aggressive, faster growth, but neglect is commonly noted as in many GBCC cases [3-4]. Since neglect is common, it is likely that it contributed to this case. However, the anonymity of the body donation program creates a limitation in drawing conclusions about the narrative history of the tumor. Information such as the duration of the disease, previous treatment, and an understanding of the patient’s decision-making is unknown. Additionally, the cause of death is an opinion of the attending physician; however, it is provided using the patient’s medical record. Since a “malignant neoplasm” was reported without qualifying it as presumed, suspected, or undetermined, it is very likely that there was clinical evidence of metastasis. Therefore, while neglect could have played a role, the focus of this case is on understanding the histopathological factors that influence the tumor growth and the role of the neoplasm in patient mortality.

Size of a BCC is a critical component to the risk of metastasis, with tumors >10 cm having a 50% incidence of metastasis [1], and only two documented cases of metastasis in a small (<1 cm) BCC [2]. Archontaki et al. did see an average GBCC duration of 14 years and attributed the large size of the tumor to duration, not an aggressive growth pattern [4]. While tumor duration is unknown in this case, the aggressive local destruction seen would likely still be accounted for by size, not duration. This is best seen in the EAC as the tumor invasion is limited to the soft tissue with intact surrounding bone (Figure 4C). The histopathological findings were consistent from multiple areas of the tumor. The characteristic BCC histology and infiltrative growth pattern were consistent throughout the tumor (Figures 2A, 4B), making it unlikely that the tumor underwent a malignant transformation during growth. However, while not a significant trend, Purnell et al. described an increase in cellular atypia in GBCC, similarly observed in this case (Figures 2B, 4A). Both the high density of mitosis and infiltrative growth pattern with areas of necrosis (Figure 4B) are common characteristics of metastatic BCC [1,3]. Additionally, high-grade cellular atypia affects prognosis in other cancers [3]; therefore, while tumor grading is not routine for BCC, cellular atypia may still play a role in prognosis.

The tumor's location also provided access to both the arteries and the veins of the left head and neck. The extensive tumor growth around multiple blood vessels could indicate hematogenous spread. Bone marrow is a common site of BCC hematogenous metastasis [1]. The presence of anemia could indicate myelophthisic anemia secondary to bone marrow invasion, which was not sampled during dissection. However, the absence of temporal bone invasion and signs of extramedullary hematopoiesis would better support chronic inflammation as the cause of the anemia. The chronic inflammatory state could also have contributed to tumor progression and spread [5]. The otorrhagia observed in the absence of middle ear or cerebrovascular damage likely resulted from soft tissue invasion of the EAC. Overall, the tumor would be considered high probability for hematogenous spread, but the lack of findings indicates that the blood vessels were more likely only used for angiogenesis.

There was no lymph node involvement found, which usually occurs in metastatic BCC. There was evidence of PNS, specifically along the facial nerve toward the stylomastoid foramen. Neoplasms do not typically exhibit passive growth along a nerve, instead growth is facilitated by some level of neural invasion. Many foci of neurotropism were observed within the carcinoma (Figure 3), which increases the likelihood of PNI elsewhere. PNI and PNS are associated with aggressive growth and extra-regional spread and thus are poor prognostic factors in head and neck cancer [2,6-7]. While this can occur independent of lymphatic spread, it increases the likelihood of lymphatic metastasis. The preference toward PNS may be due to the stromal dependence of BCC. The stromal dependence is presumed to minimize the risk of metastasis, as cells either need to migrate with a portion of their stroma or develop the ability to migrate without it [2]. PNS may provide a method for BCC growth that allows the stroma to be maintained.

The tumor exhibited many characteristics that would be high risk for metastasis without secondary proliferation of the tumor in other organs. This indicates that there was likely an interruption or inability of tumor survival outside the primary tumor, further supporting the stromal dependence of BCC. There was no specific characteristic or histopathological finding that indicated rapid growth or malignant transformation. In context with the size of BCC correlating to the metastatic rate, duration of growth likely still has a significant factor in invasiveness. While a conclusion about neglect and duration of tumor growth cannot be drawn in this case, there is no indication from a gross or histopathological standpoint why this tumor should be as large and invasive as it is.

## Conclusions

BCCs are the most common type of skin cancer, but GBCCs are exceedingly rare and aggressive. While high-risk metastatic characteristics were observed, there was no evidence of secondary neoplasms, likely indicating interruption in the process of metastasis. This further supports the stromal dependence of BCC, as the majority of invasion and damage was of soft tissue of the head and neck. Additionally, there were no specific findings that would have predisposed this tumor to the metastatic characteristics it exhibited, potentially indicating time as the causation. The lack of erosion or invasion into bone, specifically in the EAC, indicates that the tumor was not necessarily aggressive in growth, just allowed to grow for a certain, undetermined amount of time. While this specific conclusion would be limited by the anonymity of the body donation program, future research would benefit from continued literature review of the histopathological subtypes found in GBCC. Since there are a limited number of cases documented, increasing the sample size would provide a more significant conclusion about any trends of the BCC subtype(s) that develop into GBCC. The preference for PNS and the stromal dependence of BCC would be other areas that would benefit from future research efforts, particularly if there is any viability in stromal disruption in the treatment of BCC.

## References

[1] Piva de Freitas P, Senna CG, Tabai M, Chone CT, Altemani A (2017). Metastatic basal cell carcinoma: a rare manifestation of a common disease. Case Rep Med.

[2] Robinson JK, Dahiya M (2003). Basal cell carcinoma with pulmonary and lymph node metastasis causing death. Arch Dermatol.

[3] Purnell JC, Gardner JM, Brown JA, Shalin SC (2018). Conventional versus giant basal cell carcinoma, a review of 57 cases: histologic differences contributing to excessive growth. Indian J Dermatol.

[4] Archontaki M, Stavrianos SD, Korkolis DP (2009). Giant basal cell carcinoma: clinicopathological analysis of 51 cases and review of the literature. Anticancer Res.

[5] Tang L, Wang K (2016). Chronic inflammation in skin malignancies. J Mol Signal.

[6] Chen SH, Zhang BY, Zhou B, Zhu CZ, Sun LQ, Feng YJ (2019). Perineural invasion of cancer: a complex crosstalk between cells and molecules in the perineural niche. Am J Cancer Res.

[7] Medvedev O, Hedesiu M, Ciurea A (2022). Perineural spread in head and neck malignancies: imaging findings - an updated literature review. Bosn J Basic Med Sci.

